# Customer churn prediction using composite deep learning technique

**DOI:** 10.1038/s41598-023-44396-w

**Published:** 2023-10-12

**Authors:** Asad Khattak, Zartashia Mehak, Hussain Ahmad, Muhammad Usama Asghar, Muhammad Zubair Asghar, Aurangzeb Khan

**Affiliations:** 1 College of Technological Innovation, Zayed University, Abu Dhabi Campus, 144534, Abu Dhabi, UAE; 2 Institute of Computing and Information Technology, Gomal University, D.I. Khan, KP Pakistan; 3 Department of Computer Science, University of Science and Technology, Bannu, KP Pakistan

**Keywords:** Engineering, Mathematics and computing

## Abstract

Customer churn, a phenomenon that causes large financial losses when customers leave a business, makes it difficult for modern organizations to retain customers. When dissatisfied customers find their present company's services inadequate, they frequently migrate to another service provider. Machine learning and deep learning (ML/DL) approaches have already been used to successfully identify customer churn. In some circumstances, however, ML/DL-based algorithms lacks in delivering promising results for detecting client churn. Previous research on estimating customer churn revealed unexpected forecasts when utilizing machine learning classifiers and traditional feature encoding methodologies. Deep neural networks were also used in these efforts to extract features without taking into account the sequence information. In view of these issues, the current study provides an effective method for predicting customer churn based on a hybrid deep learning model termed BiLSTM-CNN. The goal is to effectively estimate customer churn using benchmark data and increase the churn prediction process's accuracy. The experimental results show that when trained, tested, and validated on the benchmark dataset, the proposed BiLSTM-CNN model attained a remarkable accuracy of 81%.

## Introduction

Customers are critical to any company's success, thus every effort is made to assure their satisfaction^1^. Maintaining the happiness of existing consumers is critical in subscription-based product expansion. The telecommunications sector is very competitive, with multiple suppliers providing comparable services. A single bad encounter can result in the irreversible loss of a consumer. Furthermore, a large migration of unsatisfied consumers could have serious financial and reputational consequences for the organization^2^.

Customer churn is the period in which a company suffers considerable losses as a result of frequent customers leaving. It is also referred to as customer attrition, and it occurs when customers stop using a company's products or services. To keep current clients, a corporation needs to analyze data in the customer database to determine the reasons for their departure^2^. The basic goal of customer churn prediction is to identify customers who are likely to leave the company. Avoiding client churn has become a critical goal for every company looking to expand its revenue. Customer turnover must be predicted in order to design effective retention measures. A corporation can take steps to retain existing customers, improve product or service quality, and avoid major losses by proactively addressing customer churn^3^.

### Research motivation

Customer churn has a substantial impact on enterprises, resulting in possible profits or losses and even the possibility of business closure^3^. Customers may transfer providers for a variety of reasons, including pricing, product delivery methods, and customer service encounters. Customer attrition can also be caused by issues such as poor product quality or a perceived lack of security. Customer churn can also be caused by dissatisfaction with present offerings or unfulfilled demands^4^. When consumers leave, businesses incur significant costs, making customer retention critical for economic viability. Anticipating customer churn through data analysis has become critical for attracting and retaining customers, since it allows firms to anticipate probable reasons for customer turnover and take early actions to resolve them.

Traditional churn prediction methods frequently have scaling concerns. For machine learning classifiers, several studies rely on human feature engineering methods. Gupta et al.^5^ used KNN for classification in previous study. However, these models do not provide an effective method for identifying clients who are likely to depart the organization.

Deep learning (DL) is a new discipline of computer science that extracts patterns from past data and makes accurate predictions using feature embedding methods. DL has been used successfully in a variety of fields, including stock price forecasting, personality recognition, disease prediction, text categorization, and others. There is considerable interest in using DL to assist companies in accurately forecasting customer churn from historical data. As a result, it is critical to conduct research and use advanced DL approaches to customer data in order to accurately assess customer churn. As a result, in our research, we present a deep learning technique called BiLSTM-CNN. BiLSTM retains useful insights from the given Telco dataset context information in both the forward and backward directions^5^. Our major goal is to effectively categorize the data as churn or non-churn using a BiLSTM-CNN model. Deep learning is used in this model to give more accurate and efficient churn prediction.

### The goal of the research

Numerous researchers^1,4,5^ have investigated the use of computational methodologies, specifically machine learning (ML) techniques, to forecast customer attrition. However, because the primary focus of these studies has been on early identification of customer turnover, they have encountered problems in dealing with the intricate interactions between predictor variables in customer churn. In this paper, we propose a novel hybrid deep learning model, BiLSTM + CNN, that combines bidirectional long/short-term memory (BiLSTM) and convolutional neural network (CNN) to predict customer turnover from a given dataset. This model aims to improve on the inadequacies of existing classifiers and forecast client turnover more accurately. We alleviate the limitation of the unidirectional LSTM layer's insufficient context information by integrating the CNN layer. The CNN layer takes BiLSTM input and ensures adequate context information, allowing effective data categorization into the churn and non-churn categories.

### Problem statement

The churn identification task is a binary classification that seeks to separate churners from non-churners. The training data is denoted by P = {p_1_, p_2_, p_3_,…, p_m_}, and the associated class label is denoted by y, where $y \in \left\{\mathrm{0,1}\right\}$. If y equals one, it denotes churn; otherwise, it represents non-churn. Our goal is to create a model that can accurately distinguish churn and non-churn based on the training data and class labels we provided.

### Research questions

*Research Questions:*

- RQ.1: How to predict customer churn from the given dataset with the use of a hybrid deep neural network model?
- RQ.2: What are the limitations of classical machine learning algorithms, and what is the efficiency of the hybrid deep neural network model as compared to classical machine learning classifiers?
- RQ.3: What is the efficiency of the proposed hybrid model for customer churn prediction concerning similar studies?

### Research contributions

This study makes the following contributions:

- A composite deep learning model is used to predict client churn.
- The efficiency of various deep learning and machine learning models for predicting client attrition.
- Evaluation of the proposed model's performance on a standard dataset for predicting customer attrition.

The remainder of the paper is structured as follows: The second portion reviews selected papers, the third section introduces the proposed technique, the fourth section analyses and assesses the results, and the last section proposes potential research avenues.

## Literature review

A literature review on Customer Churn Prediction is included in this section.

### A review of selected studies

The study conducted by^6^ investigates staff attrition through the use of several machine learning models in order to improve customer satisfaction and retention. Using the IBM dataset, five basic models and three ensembles were created and analyzed. The linear model outperformed the others in terms of accuracy, recall, and AUC. The research conducted by^7^ employs big data analysis to develop an estimating model for customer attrition in communication firms. For modelling, segmentation and regression approaches are used with good results. However, more system enhancements are required employs big data analysis to develop an estimating model for customer attrition in communication firms. For modelling, segmentation and regression approaches are used with good results. However, more system enhancements are required. Ranjan and Sood^8^ investigated the application of Twitter sentiment analysis to forecast customer attrition in Indian telecommunications. For prediction, they used the Nave Bayes classifier and TextBlob, evaluated the models with IBM SPSS, and discovered positive results for increasing customer experience and retention. However, the restricted dataset need further expansion for more robust results. Jeyakarthic et al.^9^ developed an ML-based customer churn prediction model in a cloud computing setting. With 95.50 precision, 70.49 recall, 91.71 accuracy, 95.13 F-score, and 67.20 kappa value, the model performed well. The study advises that feature selection and clustering approaches be used to improve the model further. Ahmad et al.^10^ used machine learning techniques on large amounts of data to create a client attrition prediction model for the telecom industry. The decision tree, random forest, gradient-boosted machine tree, and extreme gradient-boosted machine tree techniques were all used in the model. The XGBOOST algorithm performed the best among them. Panjasuchat et al.^11^ used supervised learning datasets to implement reinforcement learning for customer churn prediction. When the data amount was increased, DQN beat XGBoost, Random Forest, and KNN. However, when the dataset pattern changed, the performance of all methods declined. Nguyen et al.^12^ investigated customer attrition in service industries and dealt with data imbalance issues. They contrasted SMOTE and Deep Belief Network with cost-sensitive data resampling approaches, weighted loss, and focal loss. In low turnover rate conditions, focal loss and weighted loss surpassed SMOTE and DBN in prediction performance. Wahul et al.^13^ used SGD, RF, GB, AdaBoost, and Stacking classifiers to create an ensemble learning architecture for churn prediction. The stacked model outperformed individual classifiers in identifying churn consumers due to better accuracy, recall, and AUC. The researchers recommend experimenting with advanced ensemble approaches and diverse data sources. Prabadevi et al.^14^ used nine months of customer data to examine machine-learning algorithms for early customer attrition prediction. In terms of accuracy, the Stochastic Gradient Booster surpassed other methods. For hyperparameter tweaking, the study recommends employing more complex optimization approaches. Thorat et al.^15^ investigated the effectiveness of deep learning in forecasting customer attrition in the telecom business. Algorithms such as Random Forest and XGBoost were used in the study. The deep learning model deployed achieved 88% accuracy, although more data and hyperparameter optimization could improve outcomes.

Saha et al.^16^ evaluated multiple learning approaches, including CNN and ANN, using two public datasets to construct a churn prediction model. On the first dataset, CNN obtained 99% accuracy and 98% on the second. For better prediction, the study proposed utilizing structured, unstructured, and behavioral data. Seymen et al.^17^ developed ANN and CNN models for predicting retail customer attrition and compared them to various machine learning algorithms. The deep learning-based CNN model beat the others, reaching 97.62% classification accuracy. The study advises employing AI technologies to investigate missing client behavior patterns.

### Research gap and justification for using BiLSTM-CNN model for churn prediction

While basic machine and deep learning techniques have shown efficacy in customer churn prediction, earlier research have struggled to achieve greater classification accuracy levels. Incorrect parameter and layer selection can have a major impact on neural network model performance. The suggested BiLSTM + CNN model will investigate a variety of layers and parameter values to solve customer attrition in the telecoms industry. In addition, we will run further deep learning model iterations and compare their outcomes to earlier research. The proposed approach, which combines bidirectional long-term short-term memory (BiLSTM) with multiple-layer convolutional neural networks (CNN), tries to effectively identify customer turnover using accessible data.

Here are some of the reasons why the suggested BiLSTM-CNN architecture is appropriate for churn prediction:

- Bidirectional LSTM: A bidirectional LSTM has two LSTM layers: one that processes the input sequence forward and one that processes the input sequence backward. This can help the model perform better on tasks where the order of the input sequence is essential. The order of the input sequence is significant in churn prediction because it can reveal patterns that suggest whether a client is likely to churn. For example, if a customer has lately made a big number of transactions, it could signal that they are happy with the service and are less likely to churn. However, if a customer has recently cancelled their service, it may suggest that they are dissatisfied with the service and are and are more likely to churn^12^.
- Convolutional Neural Network: A convolutional neural network (CNN) is a sort of neural network that works well with sequential data. CNNs can learn to extract features from input sequences and utilize them to produce predictions. CNNs can be used in churn prediction to extract information from a customer's past data, such as their purchase history, service usage, and interactions with customer care. These characteristics can then be used to forecast if a customer is likely to churn^18^. In addition to the benefits listed above, the following are some additional advantages of employing BiLSTM-CNN for churn prediction:
- It can detect long-term dependencies in the input sequence: BiLSTM-CNN can detect long-term dependencies in the input sequence. This is significant for predicting customer turnover since customer churn is frequently driven by a set of events that occur over time. For example, a client may be unsatisfied with the service for some time before deciding to churn. These long-term dependencies can be captured by BiLSTM-CNN and used to produce more accurate predictions (20). It is relatively easy to train: BiLSTM-CNN is relatively easy to train compared to other deep learning models. This is because BiLSTM-CNN has a relatively simple architecture^19^. Overall, the BiLSTM-CNN architecture is well-suited for churn prediction since it can capture long-term dependencies in the input sequence, it is reasonably simple to train, and it has been demonstrated to be effective for churn prediction.

## Methodology

**Figure 1:**
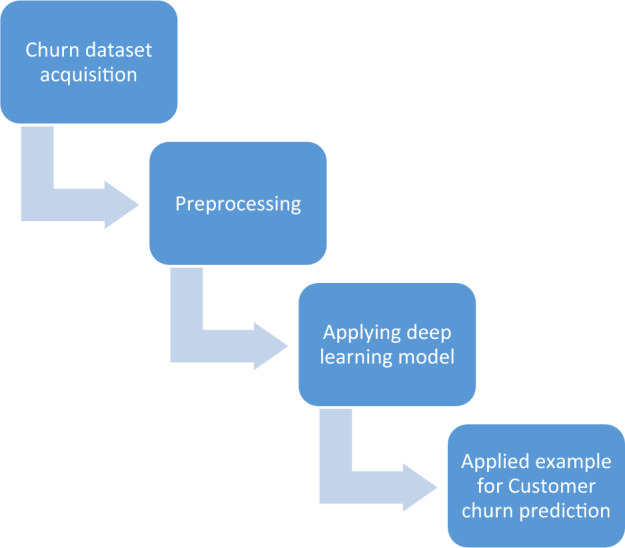
Overview of the proposed Technique.

The suggested technique is divided into following modules (Fig. 1): (i) Churn dataset acquisition, (ii) preprocessing, (iii) Applying deep learning model, and (iv) Applied example for Customer churn prediction. . Each module's specifics are provided below.

### Churn dataset acquisition

The goal of this study was to forecast client churn using telecom data and a series of calculations. We used a telecom dataset with 7033 rows and 20 different features to meet the needs of a telecom^20^. The dataset was initially partitioned into two subsets to ease the building and assessment of predictive models using the train-test-split approach from the sklearn package. This procedure produced a training dataset including 80% of the samples and a testing dataset containing the remaining 20% of the samples. To achieve a well-balanced and representative data distribution for successful model assessment, the conventional 80–20 split was used.

#### Training dataset

A training dataset is often required for the successful deployment of the suggested BiLSTM-CNN model. Typically, around 80% of the collected data is allocated for model training, though this amount may vary depending on the scenario. Datasets with appropriate labels are used to guide the hybrid neural network model during the model training phase. This labelled dataset is presented to the system in order for it to learn and improve its predicting capabilities. To use the proposed model for identifying customers who are likely to churn, data from the training dataset must be collected ahead of time. This preliminary phase guarantees that the model has the required knowledge to effectively anticipate churn for prospective clients.

#### Testing dataset

The dataset enters the model testing phase once the model training is completed. It is critical to incorporate values from the training dataset into the testing dataset in order to evaluate the model's learning performance. The enriched testing dataset is subsequently sent into the deep neural network for evaluation.

#### Organizing dataset

The dataset was obtained and put in a machine-readable ".csv" file format for the telecom corporation Telco, making it compatible with various machine learning methods.

### Data Pre-processing

The data is pre-processed extensively for the model experiment, which includes a range of feature selection strategies and pre-processing methodologies. The following list summarizes the many steps included in the pre-processing pipeline.

#### Data cleaning

Because the data we have is in string format, it cannot be used directly for further calculations. To address this, we converted the string data to numerical representation. The data had to be cleaned prior to this modification. In particular, cases where the data reflected "No Internet Service" or "No Phone Service" were correctly updated to simply "No" in multiple columns, as these entries invariably indicate the lack of the corresponding services.

#### Data encoding

Following the cleaning of textual data, we converted it into numerical form. Because the data in the target variable was in string format, we changed "Yes" entries to 1 and "No" values to 0 to facilitate numerical processing.

#### Handling missing values

Certain data points in the "Total Charges" column were discovered to be missing. To remedy this, we used 'nan' as a placeholder to replace these missing numbers. In addition, we removed empty rows and columns to ensure a more streamlined and full dataset.

#### Data transformation

We used procedures to obtain the mean and standard deviation, then scaled the results to 0 and 1, respectively, to normalize the data in the "Monthly Charges" column. We also changed the format of Boolean variables to ensure their usability in future analysis.

### Applying proposed deep learning model

**Figure 2:**
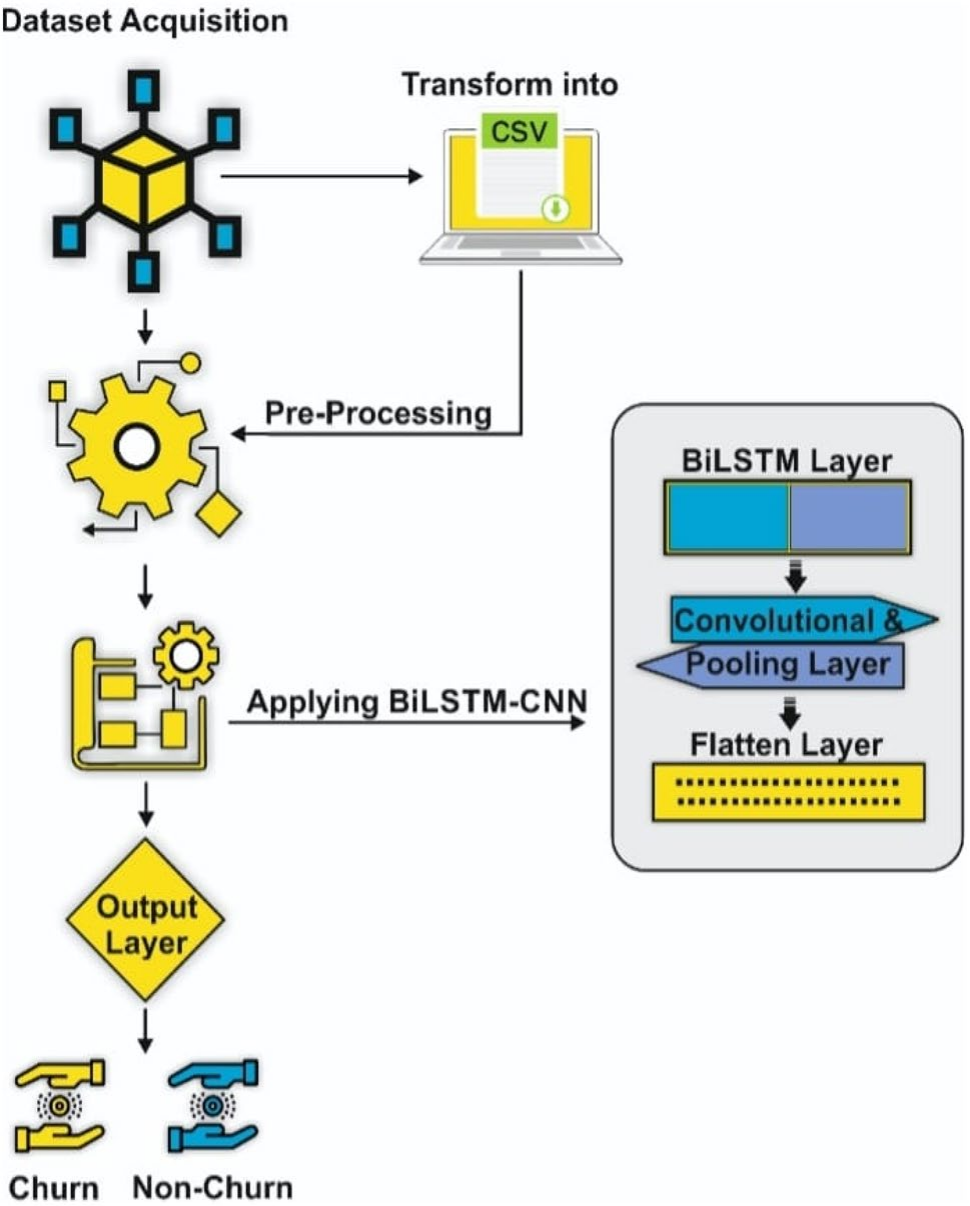
Proposed model.

Following the completion of the preprocessing work, the next phase includes the use of a deep learning model, specifically the BiLSTM + CNN architecture, to classify customer churn into discrete emotion categories. Our BiLSTM + CNN model is made up of several layers, including the Bidirectional LSTM Layer, the Convolutional Layer, the Maxpool Layer, the Flatten Layer, and the Output Layer. Figure 2 depicts a visual depiction of the detailed structure and functionality of the proposed BiLSTM + CNN model for customer churn prediction.

#### Bidirectional long short term memory (Bi-LSTM)

The BiLSTM layer shows to be really useful when applied to our dataset, which contains several features. This layer excels at collecting long-term dependencies and making access to both past (right to left) and future (left to right) contexts possible. In contrast to unidirectional LSTMs, which examine only prior information and ignore future inputs, the BiLSTM efficiently preserves the additional data essential for accurate predictions. The BiLSTM captures information from both the past and the future by merging forward and backward LSTM layers, allowing us to create accurate predictions by using the dataset's properties. The seamless incorporation of features into the BiLSTM's combined layers allows us to predict outcomes with more efficacy.

##### Forward LSTM

The pattern evaluation in the forward LSTM occurs from left to right by processing two inputs, the current "kt" and the preceding input "k_t−1_." The forward LSTM generates the corresponding output sequence $\overrightarrow{{S}_{t}}$. for an input sequence of k_1_, k_2_,…, k_i−1_.

##### Backward LSTM

To process the sequence from right to left, the backward LSTM considers two inputs: the current "k_t_" and the next input (future hidden state) "k_t+1_". The reverse LSTM generates the corresponding output sequence $\overleftarrow{{S}_{t}}$ when given an input sequence of k_i+1_,…, k_2_, k_1_.

The component total is calculated as follows: To combine the forward and backward output, use Eq. (1):

1 $$\overleftrightarrow {S_{t} } = \overrightarrow {{S_{t} }} \oplus \overleftarrow {{S_{t} }}$$

The Forward and Backward LSTM are computed using the equations below.

##### Forward LSTM equations

2 $$\overrightarrow{{m}_{t}} =\upsigma \left({H}_{m}{p}_{t}+{G}_{m}{k}_{t-1}+{U}_{m}\right)$$

3 $$\overrightarrow{{s}_{t}}=\upsigma \left({H}_{s}{p}_{t}+{G}_{s}{k}_{t-1}+{U}_{s}\right)$$

4 $$\overrightarrow {{r_{t} }} = \sigma \left( {H_{r} p_{t} + G_{r} k_{t - 1} + U_{r} } \right)$$

5 $$\overrightarrow {q\sim t} = \tau \left( {H_{q} p_{t} + G_{q} k_{t - 1} + U_{q} } \right)$$

6 $$\overrightarrow {{v_{t} }} = m_{t} \odot v_{t - 1} + z_{t} \odot v_{t}$$

7 $$\overrightarrow {{S_{t} }} = r_{t} \odot \tau \left( {v_{t} } \right)$$

##### Backward LSTM equations

8 $$\overleftarrow {{m_{t} }} = {\upsigma }\left( {H_{m} p_{t} + G_{m} k_{t + 1} + U_{m} } \right)$$

9 $$\overleftarrow {{s_{t} }} = {\upsigma }\left( {H_{s} p_{t} + G_{s} k_{t + 1} + U_{s} } \right)$$

10 $$\overleftarrow{{r}_{t}}=\sigma ({H}_{r}{p}_{t}+{G}_{r}{k}_{t+1}+{U}_{r})$$

11 $$\overleftarrow {q\sim t} = \tau \left( {H_{q} p_{t} + G_{q} k_{t + 1} + U_{q} } \right)$$

12 $$\overleftarrow {{v_{t} }} = m_{t} \odot v_{t + 1} + z_{t} \odot v_{t}$$

13 $$\overleftarrow {{S_{t} }} = r_{t} \odot \tau \left( {v_{t} } \right)$$

${m}_{t}$.is the forget gate. ${s}_{t}$. is the input gate vector. ${r}_{t}$ is the output gate vector. The output vector is ${S}_{t}$, while the cell state vector is ${v}_{t}$. Input gate weight matrices are represented by the symbols ${H}_{m}$, ${H}_{s}$, ${H}_{r}$, ${H}_{q}$. ${G}_{m}$, ${G}_{s}$, ${G}_{r}$, ${G}_{q}$ are output gate weight matrices. Bias vectors are represented by ${U}_{m}$, ${U}_{s}$, ${U}_{r}$, ${U}_{q}$. $\sigma$ show the sigmoid activation function, followed by the hyperbolic tangent function $\tau$.

Table 1 shows Symbols and their description used in BiLSTM model.

**Table 1:** Symbols used in BiLSTM.

Mathematical symbol	Description
${s}_{t}$	Input vector
${m}_{t}$	Forget gate vector
${z}_{t}$	Size of input gate vector
${r}_{t}$	Output gate vector
${S}_{t}$	Hidden state
${k}_{t-1}$	Previous hidden state
${k}_{t+1}$	Future hidden state
$\overleftrightarrow {S_{t} }$	Final representation (Element-wise sum of previous and future hidden state)
${v}_{t}$	Cell state vector
${H}_{m}$,${H}_{s}$,${H}_{r}$,${H}_{q}$	Input gate weight metrics
${G}_{m}$, ${G}_{s}$, ${G}_{r}$, ${G}_{q}$	Output gate weight metrics
${U}_{m}$,${U}_{s}$,${U}_{r}$,${U}_{q}$	Bias vectors
$\sigma$	Sigmoid activation function
$\tau$	Shows hyperbolic tangent function

#### CNN Layer

The output of the preceding BiLSTM layer is fed into the convolutional layer, making it easier to extract local n-gram features. The CNN layer is made up of several unique components, including the convolutional, maxpooling, and flatten layers, which work together to generate a comprehensive feature vector. More information about each layer is provided below:

##### Convolution

This layer performs a convolutional operation, which is a mathematical technique that combines two functions to produce a third function. A convolution filter matrix $G\in {T}^{elb}$ is used in the convolutional method. To construct the feature map, the filter matrix G is applied to each potential window over the matrix I acquired from the previous BiLSTM layer. The resulting feature map is symbolized by the letter Y.

14 $${Y}_{av}=f(D\circ {s}_{a:a+e-1,v+b-1}+B)$$

where a is 1 to (q − e + 1) and v is 1 to (m − b + 1), $B \in T$ is the bias term, “$\circ$” is the convolutional operator between G and E, and f is a nonlinear function.

We used the Relu function in this project because it outperforms other functions like tanh.

The matrix Y is obtained as follows after executing the convolution operation by using Eq. (14)

$$Y = \left[ {y_{1,1} ,y_{1,2} , \ldots y_{q - e + 1,n - d + 1} } \right],Y\in T^{q - e + 1}$$

##### Maxpool

The size of the feature map (Y) is reduced by using the maxpool layer, which selects the most significant features (maximum value). By removing non-maximal (less significant) attributes, this approach aids in optimizing computation time. The following is how the maxpool procedure is carried out:

15 $${\mathrm{B}}_{av}=\mathrm{max}({b}_{a+e-1,v+b-1})$$

The pooled feature matrix generated after using Eq. (15) is given below:

$$B=\left[{b}_{\mathrm{1,1}},b,\dots \dots .{b}_{y-e+1,h-b+1}\right],Y\in {T}^{y-e+1,h-b+1}.$$

##### Flattening

The Flatten layer converts the pooled feature map acquired in the previous step into a feature vector, ready for input into the final classification layer. Following the reshaping procedure^21^, the elements or features of the pooled feature matrix B are molded into a flattened vector, as seen below:

16 $$F=\mathrm{pooled}.\mathrm{reshape}\left[\left(y-e+1\right)\mathrm{x}\left(h-b+1\right)\right]$$

##### Classification

A single neuron-dense layer is used for prediction. The sigmoid activation function is used in this dense layer to compute the likelihood of two classes: 'churn' and 'non-churn.'The following equation (xvii) is used to calculate the net input:

17 $${T}_{in}=\sum {x}_{a}.{d}_{a}+B$$

Table 2 shows Symbols and their description used in BiLSTM model.

**Table 2:** CNN symbols.

Symbol	Description
$G$	Convolution filter matrix
$Y$	Feature map
$F$	Pooled feature matrix

The following algorithm (algorithm 1) illustrates the proposed model’s pseudo-code phases:

**Algorithm 1** Steps of BiLSTM-CNN classifier pseudo code.
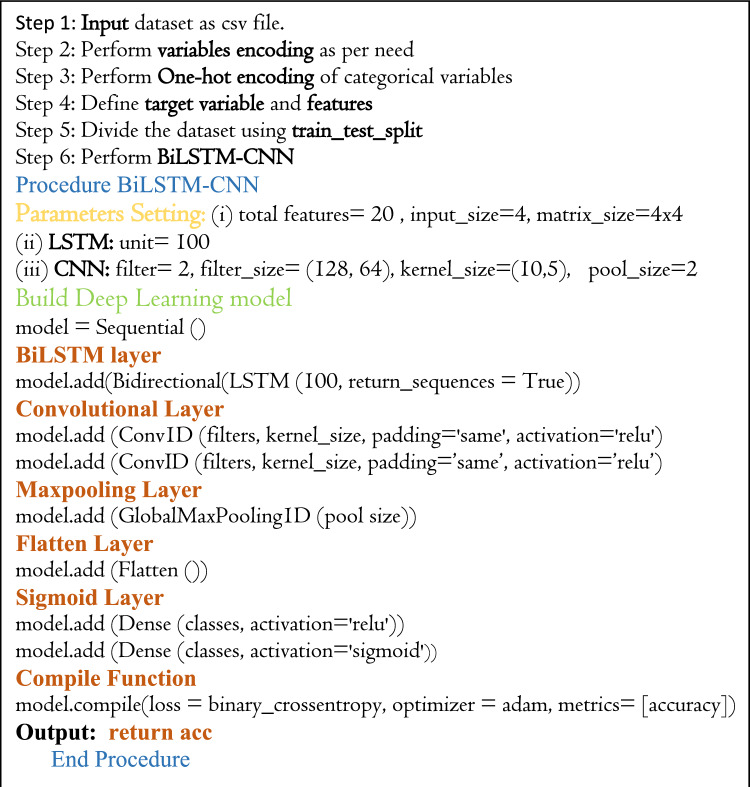


### Applied example

**Figure 3:**
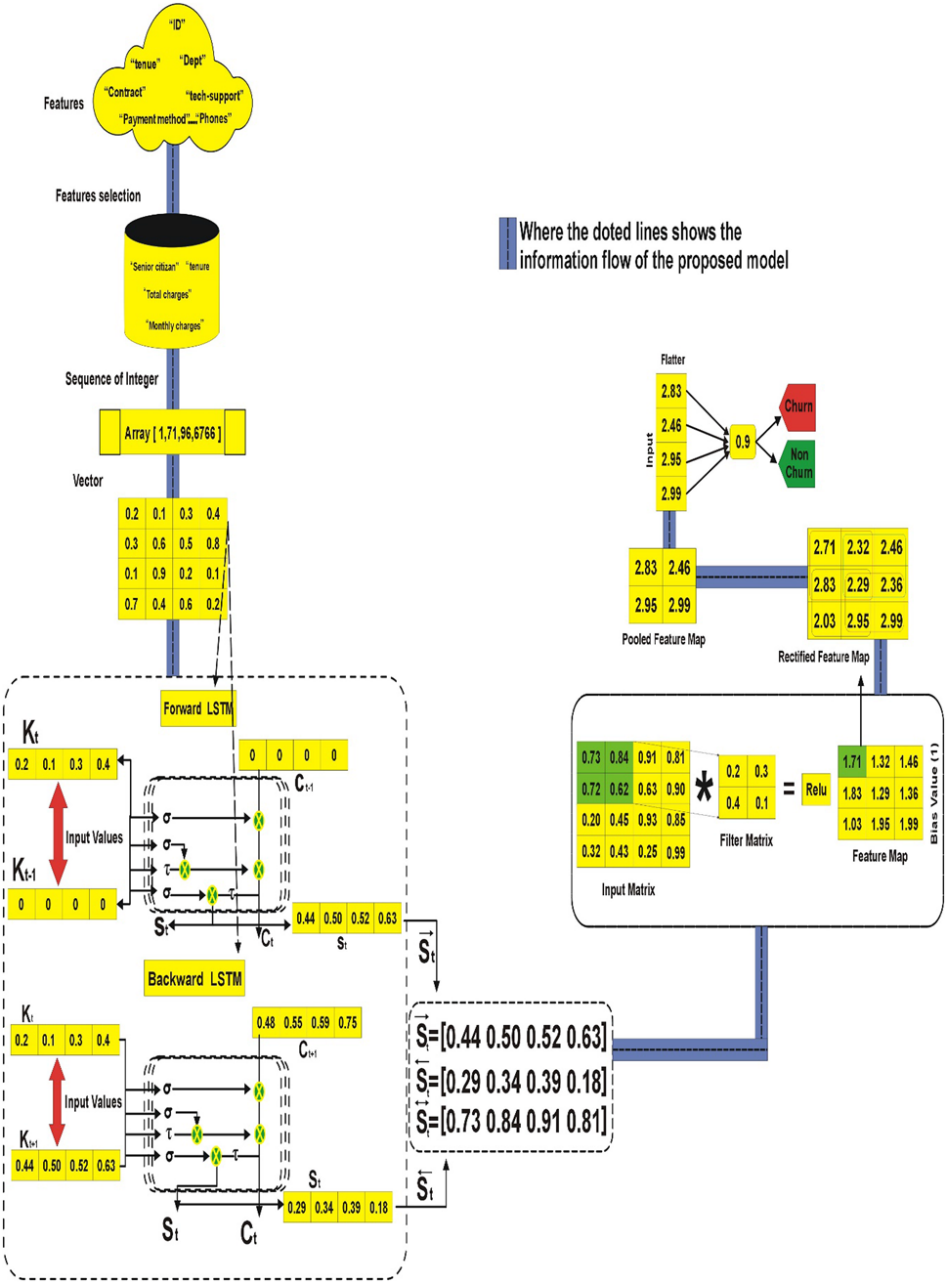
Working flow of proposed BiLSTM-CNN model.

We ran a series of simulations using existing telecoms data to estimate client turnover (See Fig. 3) The dataset for this analysis was obtained from Kaggle.com and consists of 7033 rows with 20 features such as gender, senior citizen, partner, dependents, tenure, phone service, multiple lines, internet service, online security, online backup, device protection, tech support, streaming TV, streaming movies, contract, paperless billing, payment method, monthly charges, total charges, and churn. We extracted four particular features from this dataset and fed them into the BiLSTM-CNN model. The selected characteristics were first passed through the forward and reverse LSTM layers. After that, the outputs of both BiLSTM layers were merged and transmitted to the CNN layer for further processing.

The first step in CNN is filtering. First, we align the filter matrix, and then we do element-wise multiplication of the filter matrix with the selected patch of the sentence matrix in the second step. Finally, in the third stage, we compute the feature v2 by adding all of the values from the previous phase. The second phase in the CNN process is pooling. To begin, an acceptable window size is chosen, and then a stride is decided. To optimize pooling operations, we altered the window with a threshold. The stride controls how the window moves across the rectified feature map. In the third step, the previous stage's window is used to select the maximum value, which serves as the pooling process's output. We use flattening in the third stage of CNN to turn the pooled feature matrix into a feature vector. Following that, the client is classed as churn or non-churn based on the obtained features using the CNN layer's classification algorithm. Each stage of the BiLSTM-CNN model is thoroughly investigated. We take a row from our dataset and present the step-by-step method to demonstrate the operation of the various layers in the proposed BiLSTM-CNN model for classifying the dataset as churn or non-churn.

#### Data preparation

Our approach attempts to anticipate which clients are likely to leave the organization. In our telecom dataset, "C1" represents churn, while "C2" represents non-churn cases. The dataset contains 20 features that have all been concatenated. We chose characteristics with non-zero values to prepare the data for examination. The Keras parser was used to convert the selected features into an index matrix. The matrix was then transferred to the composite deep learning (DL) model's embedding layer. The grading criteria include "SeniorCitizen," "tenure," "MonthlyCharges," and "TotalCharges." Each of these characteristics is transformed into a vector of floating-point values. A scalar embedding would be something like [1, 71, 96, 6766]. The matrix packaging produced the following result: [0.2, 0.1, 0.3, 0.4], [0.3, 0.6, 0.5, 0.8], [0.1, 0.9, 0.2, 0.1], [0.7, 0.4, 0.6, 0.2]. These values indicate the embeddings of the selected characteristics as streaming numbers.

#### Contextual information extraction

Unidirectional LSTM can only access information from the prior context, which may not be sufficient for certain categorization tasks that require knowledge from both past and future contexts. To overcome this, we implemented a bidirectional architecture that makes use of both forward and backward LSTM. This enables us to process the sequence in both directions, acquiring extensive contextual information from the entire sequence, including elements from the past and future. The preceding neural network layer's standardized feature map is utilized as input for this layer. The new candidate value (q–t), output gate (rt), forget gate (mt), and input gate (st) are the primary components of BILSTM layer calculations.

#### Hidden layer 1

The current input (kt) and the previous state (S_t−1_)) of the LSTM are used in the computation. The calculation is governed by Eqs. (1)–(6). Finally, as the output of the first hidden layer, which corresponds to the forward pass LSTM, the hidden state ($\overrightarrow{{S}_{t}}$ ) is obtained.

$$\mathop {\left[ {\begin{array}{*{20}c} {0.44} \\ {0.50} \\ {0.52} \\ {0.63} \\ \end{array} } \right]}\limits^{{\overrightarrow {{S_{t} }} }} = \mathop {\left[ {\begin{array}{*{20}c} 1 \\ 1 \\ 1 \\ 1 \\ \end{array} } \right]}\limits^{{r_{t} }} \odot \tau \mathop {\left( {\begin{array}{*{20}c} {0.48} \\ {0.55} \\ {0.59} \\ {0.75} \\ \end{array} } \right)}\limits^{{v_{t} }}$$

#### Hidden layer 2

The LSTM's current input (k_t_) and future state (k_t+1_) are used in the computation. The calculations are carried out using Eqs. (7)–(12). Finally, the hidden state ($\overleftarrow{{S}_{t}}$) is computed as the output of the second hidden layer, which corresponds to the LSTM backward pass.

$$\mathop {\left[ {\begin{array}{*{20}c} {0.29} \\ {0.34} \\ {0.39} \\ {0.18} \\ \end{array} } \right]}\limits^{{\overleftarrow {{S_{t} }} }} = \mathop {\left[ {\begin{array}{*{20}c} {0.29} \\ {0.34} \\ {0.39} \\ {0.18} \\ \end{array} } \right]}\limits^{{r_{t} }} \odot \tau \mathop {\left[ {\begin{array}{*{20}c} {0.29} \\ {0.34} \\ {0.39} \\ {0.18} \\ \end{array} } \right]}\limits^{{v_{t} }}$$

#### BiLSTM outcome

Finally, using Eq. (1), the forward and backward LSTMs are merged to provide the final result, which can be written as follows:

$$\mathop {\left[ {\begin{array}{*{20}c} {0.73} \\ {0.84} \\ {0.91} \\ {0.81} \\ \end{array} } \right]}\limits^{{\overleftrightarrow {S_{t} }}} = \mathop {\left[ {\begin{array}{*{20}c} {0.44} \\ {0.50} \\ {0.52} \\ {0.63} \\ \end{array} } \right]}\limits^{{\overrightarrow {{S_{t} }} }} \oplus \mathop {\left[ {\begin{array}{*{20}c} {0.29} \\ {0.34} \\ {0.39} \\ {0.18} \\ \end{array} } \right]}\limits^{{\overleftarrow {{S_{t} }} }}$$

#### CNN layer

**Figure 4:**
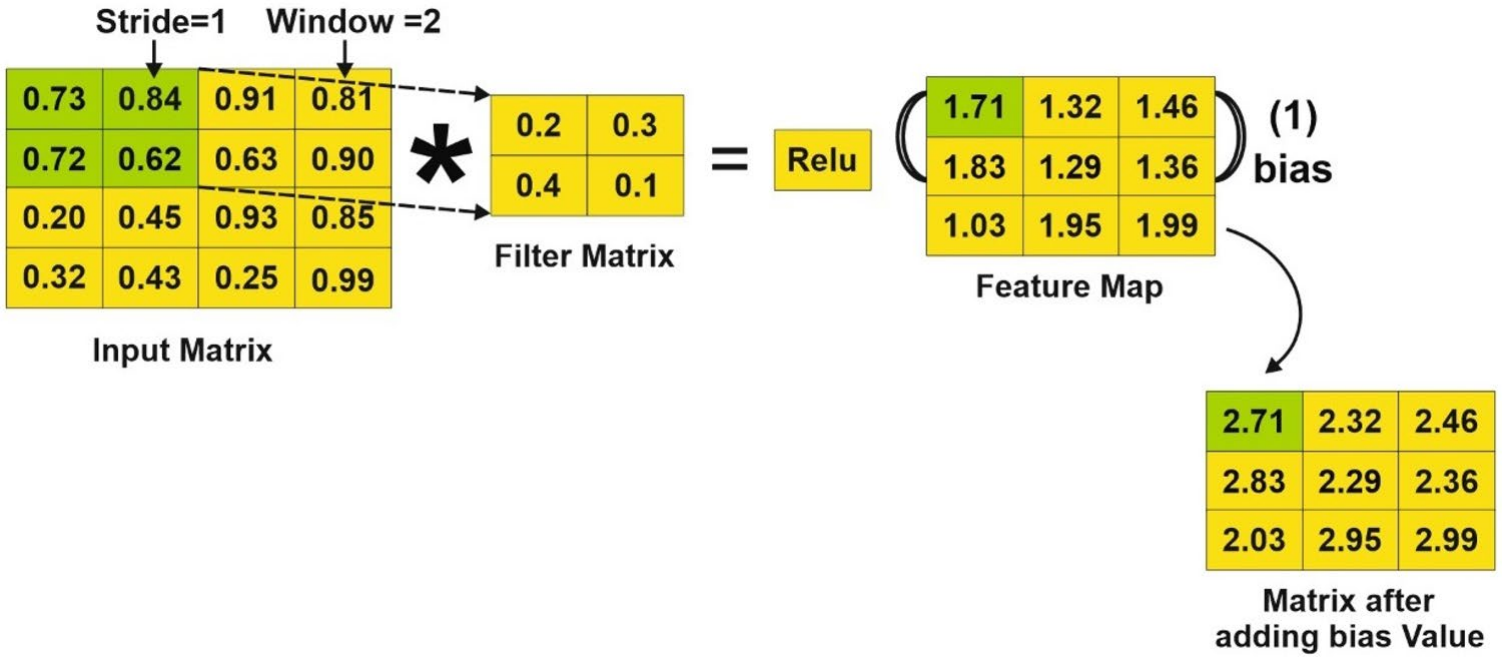
Convolution operation.

To extract local features, the convolutional layer uses the input from the preceding BiLSTM layer. The following is how the feature extraction method is carried out:

*Step-1*: Filtering:

As shown in Eq. (14), the filter matrix is convolved over the input matrix during the filtering stage to produce the convolved feature map. This procedure, depicted in Fig. 4, consists of three steps. In the first phase, the filter matrix is aligned, followed by element-wise multiplication of the filter matrix with the sentence matrix patch of choice in the second step. Finally, the feature v2 is computed in the third step by aggregating all of the information obtained in the previous phase.

**Figure 5:**
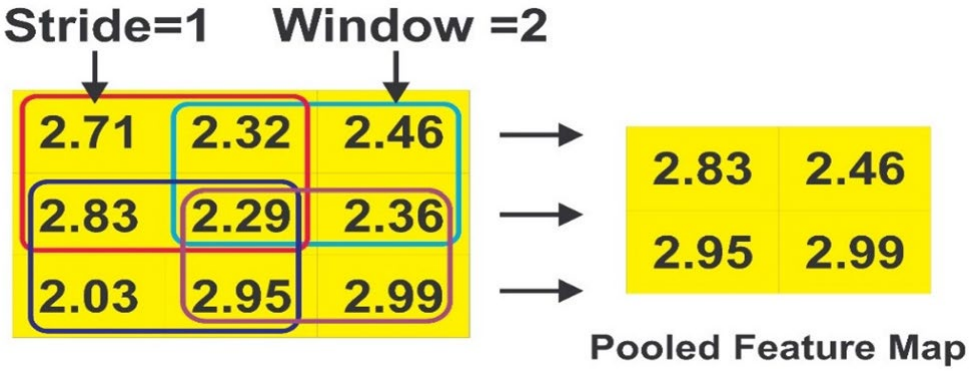
Pooling operation.

*Step-2*: Pooling:

We used Eq. (15) for reducing the convolved feature map obtained in the previous stage. The pooling procedure is depicted in Fig. 5 as three steps: The first step is to choose a proper window size. The second step is to determine the stride by adjusting the window with a threshold. The stride specifies how far the window moves across the rectified feature map. In the third phase, the previous stage's window is used to select the maximum value, which acts as the pooling process's output.

**Figure 6:**
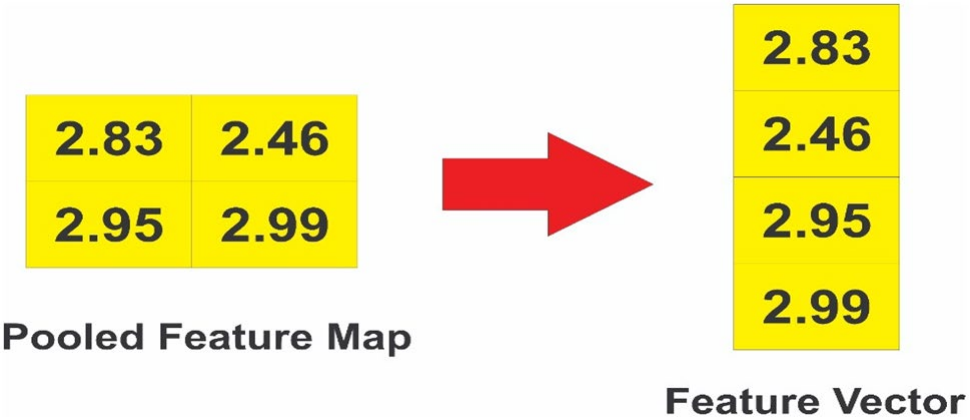
Flattening layer.

*Step-3*: Flattening:

Equation (16) is used to convert the pooled feature matrix acquired in the previous step into a feature vector, as shown in Fig. 6.

#### Classification layer

The feature vector from the previous CNN layer is supplied into the classification layer, which calculates the class probability using the sigmoid function. The net input of the BiLSTM-CNN model can be estimated using Eq. (17) as follows:

$$\begin{gathered} {\text{T}}_{{{\text{in}}}} = {\text{x}}_{{1}} .{\text{d}}_{{1}} + {\text{x}}_{{2}} .{\text{d}}_{{2}} + {\text{x}}_{{3}} .{\text{d}}_{{3}} \ldots {\text{x}}_{{\text{n}}} .{\text{d}}_{{\text{n}}} + {\text{B}} \hfill \\ {\text{T}}_{{{\text{in}}}} = {2}.{83}*0.{6} + {2}.{46}*0.{5} + 2.{95}*0.{8} + {2}.{99}*0.{9} + \left( {0.{8}} \right) \hfill \\ {\text{T}}_{{{\text{in}}}} = {1}.{698} + {1}.{23} + {2}.{36} + {2}.{691} + 0.{8} \hfill \\ {\text{T}}_{{{\text{in}}}} = {8}.{779} \hfill \\ \end{gathered}$$

L = F(T_in_), where L is the output, F is the sigmoid activation function, while T_in_ stands for net input.

The following is the procedure for calculating L:

$$\begin{gathered} {\text{L}} = {1}/\left( {{1 } + {\text{ e}}^{{ - {\text{Tin}}}} } \right) \hfill \\ \;\;\;{ = 1}/ \, \left( {{1 } + {\text{ e}}^{{ - {8}.{779}}} } \right) \hfill \\ \;\;\;{ = 1}/\left( {{1 } + \, 0.000{1}} \right) \hfill \\ \;\;\;{ = 1}/{1}.000{1} \hfill \\ \quad = 0.{9} \hfill \\ \end{gathered}$$

#### Decision rule

$$f\left( x \right) = \left\{ \begin{gathered} 1\quad \left( {Churn} \right), L > 0.5 \hfill \\ 0\quad \left( {Non - Churn} \right), otherwise \hfill \\ \end{gathered} \right.$$

L = 0.9 > 0.5, using the above decision rule.

**Figure 7:**
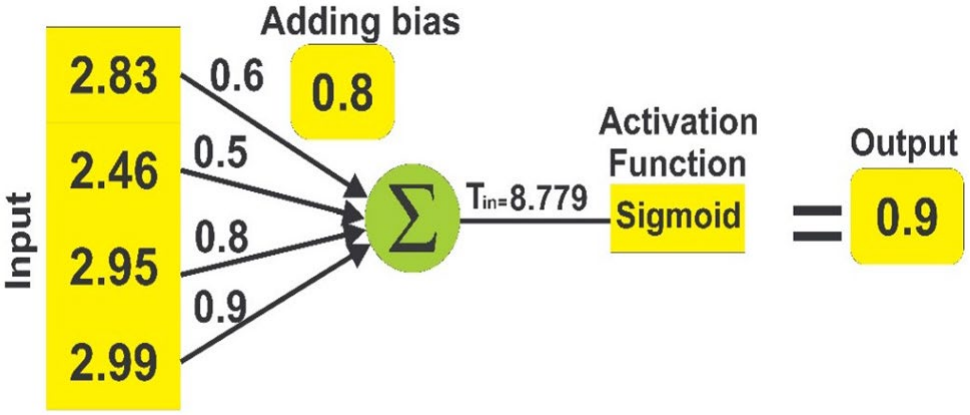
Final classification.

Our input is projected to be churn, as shown in the Fig. 7 below:

As seen in Fig. 7, the predicted outcome is “churn”.

## Result and discussion

This chapter covers the experimental setup, the results acquired, and the analysis of those results.

### Response to the initial research question

The initial research question is:

"How to predict customer churn from a given dataset by using a hybrid deep neural network model"

We used gender, SeniorCitizen, Partner, Dependents, tenure, PhoneService, MultipleLines, InternetService, OnlineSecurity, OnlineBackup, DeviceProtection, TechSupport, StreamingTV, StreamingMovies, Contract, PaperlessBilling, PaymentMethod, MonthlyCharges, TotalCharges, and churn from the customer's dataset. These characteristics were fed into the BiLSTM-CNN model, which predicted which customers were likely to leave the telecom service. We ran several experiments using these characteristics to improve the performance of the hybrid model. Table 3 shows the various parameters of the proposed BiLSTM-CNN model.

**Table 3:** Parameter setting of the proposed system.

Parameter	Value
Sample size	7033
Total features	20
No of convolution layers	2
No of dense	2
BiLSTM unit size	20, 50, 60, 60, 80, 60, 60, 100, 50, 90
Activation function	Sigmoid
Optimizer	Adam
Dense size	20, 1
No of epochs	10
Batch size	32

We investigated several parameter choices for the variables in the BiLSTM-CNN model through a series of tests, measuring test loss, accuracy, and training time. Table 4 displays the results of the BiLSTM-CNN model, including training duration, test loss, and test accuracy. Among the several BiLSTM-CNN models, the "BiLSTM-CNN (10)" model shines out, with filter sizes of 8 and 10, a number of filters of 60 and 50, and a BiLSTM unit length of 90 neurons, attaining an amazing accuracy of 81%. The various models were ranked based on their test accuracy, which ranged from 72 to 80%. Table 4 displays these data in ascending order.

**Table 4:** Efficiency of the proposed models.

Name of model	Accuracy (%)	Test loss	Training time (s)
(1) BiLSTM-CNN	0.72	0.46	1
(2) BiLSTM-CNN	0.74	0.46	1
(3) BiLSTM-CNN	0.75	0.44	3
(4) BiLSTM-CNN	0.75	0.46	1
(5) BiLSTM-CNN	0.76	0.45	1
(6) BiLSTM-CNN	0.77	0.46	1
(7) BiLSTM-CNN	0.77	0.44	1
(8) BiLSTM-CNN	0.78	0.41	1
(9) BiLSTM-CNN	0.80	0.40	1
(10) BiLSTM-CNN	0.81	0.40	2

#### Precision, recall and F1 score of BiLSTM-CNN

**Figure 8:**
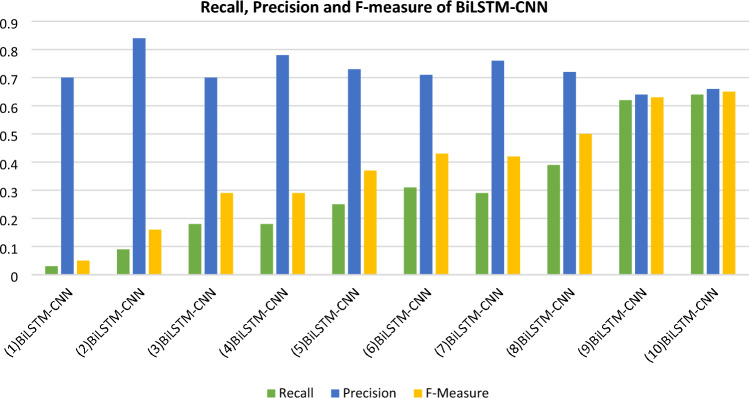
Recall, precision and F-measure of BiLSTM-CNN models.

The accuracy, recall, precision, and f-score values of various BiLSTM-CNN models are shown in Table 5. Figure 8 also shows the evaluation measures for the BiLSTM-CNN models, such as precision, recall, and f-score. The recall, precision, and f-score values are represented on the X-axis, while the equivalent metrics for each of the BiLSTM-CNN models are shown on the Y-axis.

**Table 5:** Precision, Recall and F1 score of BiLSTM-CNN models.

Name of model	Recall	Precision	F-measure
(1) BiLSTM-CNN	0.03	0.70	0.05
(2) BiLSTM-CNN	0.09	0.84	0.16
(3) BiLSTM-CNN	0.18	0.70	0.29
(4) BiLSTM-CNN	0.18	0.78	0.29
(5) BiLSTM-CNN	0.25	0.73	0.37
(6) BiLSTM-CNN	0.31	0.71	0.43
(7) BiLSTM-CNN	0.29	0.76	0.42
(8) BiLSTM-CNN	0.39	0.72	0.50
(9) BiLSTM-CNN	0.62	0.64	0.63
(10) BiLSTM-CNN	0.64	0.66	0.65

The mathematical equations Eqs. (18), (19) and (20) determine the recall, precision, and f-score, respectively. Figure 8 depicts a comparison of precision, recall, and f-score for the several variant models.

18 $$Recall= \frac{Churn\;Correctly\;Identified}{Churn\;correctly\;identified+Churn\;Incorrectly\;labeled\;as\;Non-Churn}$$

19 $$Precision=\frac{Churn\;Correctly\;identified}{Churn\;correctly\;identified+indiviual\;incorrectly\;labeled\;as\;Churn}$$

20 $$F-measure=2*\frac{precision*recall}{precision+recall}$$

### Response to the second research question

**Figure 9:**
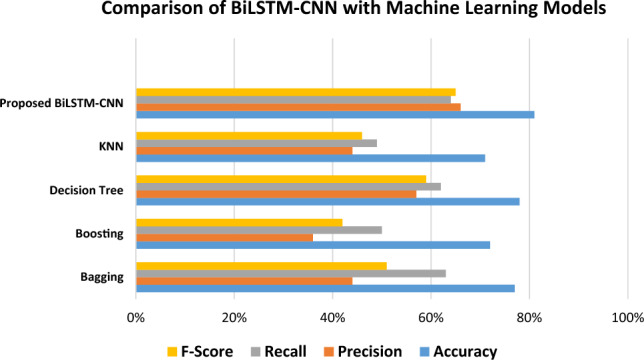
Comparison of BiLSTM-CNN with machine learning models.

The suggested BiLSTM-CNN model's performance for customer churn prediction was thoroughly examined and compared to numerous traditional machine learning models (see Fig. 9). This comparison was carried out to answer the second research question, which investigates the limitations of classical machine learning methods and compares the efficiency of the hybrid deep neural network model to traditional machine learning classifiers.

Table 6 compares the accuracy of the machine learning models to the proposed BiLSTM-CNN model.

**Table 6:** Comparison with ML methods.

Study	Models	Accuracy (%)	Precision (%)	Recall (%)	F-score (%)
Li and Zhou^7^	SVM	79	48	69	57
Manghnani et al.^22^	Bagging	77	44	63	51
Sari et al.^23^	Boosting (AdaBoost)	72	36	50	42
Herdian et al.^24^	Decision Tree	78	57	62	59
Gupta et al.^5^	KNN	71	44	49	46
Proposed	BiLSTM-CNN	81	66	64	65

#### SVM (Support Vector Machine) vs. BiLSTM-CNN (Proposed)

In comparison to the suggested model, SVM performed poorly in customer churn prediction, reaching an accuracy of only 76%. SVM is not well-suited for huge datasets, and its performance worsens when target classes overlap. Furthermore, SVM needs feature scaling, which might result in decreased accuracy. Furthermore, SVM performs badly when the number of attributes for each data sample exceeds the number of training data samples.

#### Ensamble (Begging and Boosting) vs. BiLSTM-CNN (Proposed)

In terms of customer turnover prediction, XBG attained a comparatively low accuracy of 79%. This reduced accuracy can be attributed to the fact that the XGB classifier takes a long time to learn and score. Furthermore, the limitation of only working with numerical features may limit its efficacy. Furthermore, if hyperparameters are not properly set, XGB can be prone to overfitting, reducing its predictive ability even further.

#### Decision tree vs. BiLSTM-CNN (Proposed)

In comparison to the proposed model, the decision tree performed poorly, with an accuracy of 78%. Due to the intrinsic intricacy of its calculations, decision trees often necessitate lengthier training periods. Furthermore, decision trees can become highly sophisticated, and even minor changes in the input might result in the formation of a whole new tree, making them potentially unstable. Furthermore, decision trees are ineffective when dealing with continuous numerical variables.

#### KNN (K Nearest Neighbor) vs. BiLSTM-CNN (Proposed)

The suggested BiLSTM-CNN model was compared to the KNN model. KNN performed poorly in predicting customer attrition, with an accuracy of only 71%. KNN's shortcomings stem from its inability to handle bigger imbalanced datasets and its vulnerability to noisy data, missing values, and outliers, resulting in unsatisfactory accuracy. In contrast, the suggested BiLSTM-CNN model addresses classical machine learning difficulties such as manual feature extraction, restricted hyperparameter tuning capacity, and poor performance on large datasets. We can obtain better attrition prediction in the telecoms sector with higher accuracy and less processing time by using BiLSTM-CNN, making it a better alternative for customer churn prediction than KNN.

#### Algorithm complexity of the proposed model

A BiLSTM-CNN model for churn prediction can be defined in terms of time and space complexity.

##### Time complexity

The number of operations executed during the training and prediction phases determines the time complexity of a BiLSTM-CNN model for churn prediction. The LSTM and CNN layers are the most significant contributors to time complexity. *LSTM Time Complexity*: A single LSTM cell has a time complexity of O(n), where n is the number of input features to the LSTM cell. This complexity is twice for a BiLSTM layer since it processes input in both forward and backward directions. The LSTM layer's overall time complexity is O(T * n), where T is the number of time steps (sequence length) in the input data. *CNN Time Complexity:* The time complexity of a 1D Convolutional Neural Network (CNN) layer is determined by the kernel size, the number of filters (channels), and the length of the input sequence. Convolution takes O(k * n) operations for each filter, where k is the kernel size. The overall time complexity of the CNN layer is O(F * k * n) if there are F filters. *Overall Time Complexity:* The total temporal complexity of the BiLSTM-CNN model is the sum of the complexities of the BiLSTM and CNN layers, which can be written as: O(T * n) + O(F * k * n).

##### Space complexity

The number of parameters in the BiLSTM-CNN model, which includes weights, biases, and other trainable parameters, determines the space complexity of the model. Space Complexity of the BiLSTM layer is O(L * (4 * n * n + 4 * n)), where L is the number of LSTM cells (units) in the layer. CNN layer has a space complexity of O(F * (k * n + 1)), where F is the number of filters, k is the size of the kernel, and n is the number of input features. The BiLSTM-CNN model's overall space complexity is the sum of the space complexities of the BiLSTM layer and the CNN layer, which can be expressed as: O(L * (4 * n * n + 4 * n)) + O(F * (k * n + 1)).

### Response to the Third Research Question:

To answer the final RQ, "How effective is the proposed hybrid model for predicting customer churn in comparison to other DL models and similar studies?" We ran a series of tests to evaluate the effectiveness of the proposed BiLSTM-CNN for churn prediction to the findings of other studies.

#### Comparison with Similar studies

Table 7shows the comparison of similar studies with the proposed model.

**Table 7:** Comparison with baseline study.

Study	Model	Accuracy
Gupta et al.^5^	KNN	71%
Proposed	BiLSTM-CNN	81%

Gupta et al.^5^ used KNN model on customer turnover prediction, achieving and accuracy of 71% In comparison, our suggested model, BiLSTM-CNN, beat all of these feature selection approaches, as well as many other machine learning models, with an outstanding accuracy of 81% in predicting customer turnover.

#### Comparison with other DL models

The study evaluated the BiLSTM + CNN model to other DL approaches such as CNN, RNN, and BiLSTM for predicting customer churn using prior customer data. The results are summarized in Table 8.

**Table 8:** The suggested model (BiLSTM + CNN) versus other deep learning models.

Deep learning model	Accuracy (%)	Precision (%)	Recall (%)	F-score (%)
CNN	75	64	63	64
RNN	76	62	54	63
BiLSTM	72	61	63	61
Proposed Model (BiLSTM-CNN)	81	66	64	65

- CNN VS Proposed BiLSTM-CNN
  The goal of this experiment was to compare the effectiveness of the proposed BiLSTM + CNN model to a mono CNN model. Based on the results in Table 8, it is clear that the CNN model performed the worst in terms of precision, recall, F1-score, and accuracy. The CNN model's lower ranking can be attributable to two major factors: (i) its failure to maintain text sequence contextual information, and (ii) its need for a big dataset to improve classifier performance.
- RNN VS Proposed BiLSTM-CNN
  The aim of this study was to assess the effectiveness of the proposed BiLSTM + CNN model in comparison to an RNN model. As shown in Table 8, the RNN model exhibited inferior performance in terms of precision, recall, F1-score, and accuracy. The shortcomings of recurrent neural network models lie in their inability to retain information over extended time frames, as they struggle with managing excessively long patterns. Consequently, the RNN model demonstrated unsatisfactory performance^3^.
- BiLSTM VS Proposed BiLSTM-CNN
  The purpose of this study was to compare the effectiveness of the suggested BiLSTM + CNN model to a regular BiLSTM model. As shown in Table 8, the BiLSTM model performed the worst in terms of accuracy, recall, F1-score, and precision. BiLSTM models' shortcoming is their capacity to retain just past context information while ignoring future context information, which may provide a more comprehensive comprehension of the context in the reviewed text. As a result, it fared the worst of all the models tested.

## Conclusion and future work

To detect clients who are vulnerable to churn, this study applies a deep neural network dubbed BiLSTM-CNN. The following elements are included in the proposed BiLSTM-CNN model: (i) Collection of datasets; (ii) Preparation of datasets; (iii) Feature representation; (iv) Feature concatenation; and (v) Classification. When compared to the baseline procedures, the results show enhanced accuracy (81%), precision (66%), recall (64%), and f-score (65%).

### Limitations

This study has certain limitations, which are listed below: (i) it focused only on binary classification; (ii) The research will only use the BiLSTM-CNN model, without experimenting with other composite Deep Learning models; (iii) Only numeric features will be used to predict customer attrition; (iv) The study will only use random feature embedding, without considering alternative representation models such as "pre-trained"; and (v) Given the dataset contains 20 features, more than twenty features should be used.

### Future work

The following study directions are suggested for the future: (i) To improve performance, researchers should conduct experiments on large-scale datasets and consider expanding the dataset to include varied domains. (ii) Future research should look into multidimensional CNN approaches and other deep neural network models for predicting customer churn. (iii) In future research, the suggested BiLSTM-CNN model should be used to tackle multiclass issues, multi-label classification, and imbalanced classification. (iv) Beyond numeric characteristics, more sorts of features should be researched to produce more efficient results. (v) In future research endeavors, pre-training feature embedding should be examined as a feasible technique.

## Data Availability

Underlying data supporting the results can be provided by sending a request to the corresponding/submitting author.
